# Dyspnea is a dangerous symptom in the pre-hospital setting

**DOI:** 10.1186/1757-7241-23-S2-O7

**Published:** 2015-09-11

**Authors:** Morten T Bøtker, Hans Kirkegaard, Erika F Christensen, Christian J Terkelsen

**Affiliations:** 1 Research Department, Prehospital Emergency Medical Service, Aarhus, Denmark; 2 Research Center for Emergency Medicine, Aarhus University Hospital, Aarhus, Denmark; 3 Department of Cardiology B, Aarhus University Hospital, Aarhus, Denmark

## Background

Electrocardiogram (ECG) based telemedicine is a cornerstone in pre-hospital triage of patients with suspected ST-elevation myocardial infarction (STEMI). An ECG transmitted from the ambulance is reviewed by a cardiologist on-call in case of ongoing or recent chest pain, resuscitation from cardiac arrest, acute dyspnea of unknown origin and other suspicion of STEMI. We hypothesize that unresolved dyspnea is an independent predictor of mortality in this prehospital setting and that the mortality is higher in patients with acute dyspnea of unknown origin than in patients with chest pain.

## Methods

Population based follow-up study. We included patients triaged using ECG based telemedicine in the Central Denmark Region from June 1, 2008 to January 1, 2013 in our analyses. Mortality-data was obtained from the Danish Civil Registration System. Since survival curves did not fulfill the proportional hazards assumption, Cox proportional hazards regression was waived. Instead, to determine relative risks, we used a generalized linear regression model using pseudo-observations.

## Results

A total of 17,361 patients were triaged by use of ECG based telemedicine. The indication was chest pain in 12,204 (70%) of the patients, acute dyspnea of unknown origin in 1,461 (8 %), resuscitated from cardiac arrest in 163 (1%) and other suspicion of STEMI in 3,533 (20%). When adjusting for age, sex, systolic blood pressure and Charlson Comorbidity Index (p<0.001), 30-day mortality was higher in patients with unresolved dyspnea than in patients with chest pain with a RR 2.55 (CI 2.09-3.10). This difference remained significant at 4 years with a RR of 1.34 (CI 1.24-1.45).

## Conclusion

Acute dyspnea of unknown origin in the pre-hospital setting is an independent predictor of mortality and the mortality is higher than in patients with chest pain. Future research should focus on possibilities for improving early diagnosis and treatment of these patients.

